# Supplementary material for: Enhancing epidemic forecasting with a physics-informed spatial identity neural network
Source: PLoS One. 2025 Sep 15;20(9):e0331611. doi: 10.1371/journal.pone.0331611 (PMC12435659; doi:10.1371/journal.pone.0331611)
Supplement: Attachment — Submitted filename: Response to Reviewers.docx [file pone.0331611.s002.docx]

# Note from the authors

We highly appreciate the reviewer’s thoughtful comments, which have helped us to improve the clarity and rigor of the manuscript. We would also like to express our gratitude to the editorial team for facilitating a constructive review, and for their support in the submission process.

**Please note that all changes to the manuscript were made in the track-change mode, in order to facilitate further review. Row numbers provided below correspond to the track-changed version of the manuscript, in a Simple Markup view.**

# Reviewer #1

1- Add numeric values for model efficiency metrics to the abstract.

**Reply:**

Thank you for your valuable comment. In response, we have added the model size and computation time observed during the experiments to the abstract to help readers better understand the efficiency of the proposed model.

**Changes in the manuscript:**

Information added in the manuscript: Abstract section, rows 22-23.

2- Using research papers published in 2025 and adding them to the related works.

**Reply:**

Thank you very much for your suggestion. In response, we have updated the "Related works" section by incorporating the following research papers published in 2025 on infectious disease prediction. These papers address the application of machine learning models to real-world data, as well as newly proposed spatio-temporal graph neural network models.
These additions help ensure the timeliness and relevance of the referenced literature.

- Battineni G, Chintalapudi N, Amenta F. Forecasting of COVID-19 epidemic size in four high hitting nations (USA, Brazil, India and Russia) by Fb-Prophet machine learning model. Applied Computing and Informatics. 2025;21.1/2: 2-11.
- Sadig HE, Kamal M, ur Rehman M, Habadi MI, Alnagar DK, Yusuf M, et al. Advanced time complexity analysis for real-time COVID-19 prediction in Saudi Arabia using LightGBM and XGBoost. Journal of Radiation Research and Applied Sciences. 2025;18.2: 101364.
- Luo J, Wang X, Fan X, He Y, Du X, Chen YQ, et al. A novel graph neural network based approach for influenza-like illness nowcasting: exploring the interplay of temporal, geographical, and functional spatial features. BMC Public Health. 2025;25.1: 408.
- Wang X, Jin Z. Multi-region infectious disease prediction modeling based on spatio-temporal graph neural network and the dynamic model. PLOS Computational Biology. 2025;21.1: e1012738.

**Changes in the manuscript:**

Information added in the manuscript: Related works section, rows 101-105, 119-122.

3- Explaining the proposed algorithm in clear and simple steps for easy follow-up.

**Reply:**

We appreciate your insightful comment. In response, we recognized that the explanation of the processing within the SIR module in the Methodology section was particularly complex and difficult to follow. Therefore, we have revised the structure of the description to improve the clarity of the reasoning process. Additionally, to help readers better understand the overall inference flow, we have included pseudocode for the proposed algorithm.

**Changes in the manuscript:**

Description revised in the manuscript: Methodology section, rows 185-187, 189-195, 197-199.

Information added in the manuscript: Methodology section, rows 205.

4- Discuss tables and graphs better and reflect a full understanding of what is happening.

**Reply:**

Thank you for your suggestion. In response, we have carefully revised the descriptions of the experimental results in the Experimental study section. Specifically, we have enhanced the explanations accompanying each table and figure to clarify what each result shows and how they can be interpreted. We aimed to ensure that the interpretations are logically grounded and clearly connected to the data, so that readers can better understand the implications of the results without ambiguity.

**Changes in the manuscript:**

Description revised in the manuscript: Experimental study section, rows 278-280, 284-300, 314-316, 317-320, 338-343, 363-366, 368, 370-374.

5- For conclusions not based on direct numerical values and future work not sufficiently explained.

**Reply:**

Thank you very much for pointing this out. To address the concern regarding the lack of direct numerical evidence in the conclusion, we have revised the section to include quantitative results. Specifically, we now mention that the proposed model ranked within the top two across all forecasting scenarios in terms of MAE, and that its encoder architecture achieved an average improvement of 7.4% in MAE and 5.8 % in RMSE compared to the second-best baseline encoder. These additions are intended to more clearly convey the benefits of our model from a quantitative perspective.

Regarding the future work section, we have refined the description to provide a more concrete direction. We now specify the types of external data we plan to incorporate—such as mobility patterns, distributions of viral strains, and vaccination rates—and outline our modeling strategies, including the use of dynamic graph structure learning and extensions of the SIR module. These revisions aim to clarify how we intend to enhance the model’s ability to capture epidemic dynamics and support public health decision-making.

**Changes in the manuscript:**

Description revised in the manuscript: Conclusion section, rows 435-437, 440-441, 443-452.

# Reviewer #2

This study proposes a physics-informed spatial identity neural network for time-series forecasting of epidemic cases. The method integrates spatiotemporal information with domain knowledge for forecasting, and its performance is demonstrated using real data collected from Japan and the USA. The following comments are provided for the authors’ consideration:

1. The prediction performance heavily depends on the partitioning of datasets, and the proposed method is primarily applicable to time-series data with stationary patterns. This is evident from the results in Table 1 and Table 2. The proposed method does not consistently outperform others in all cases. The authors should highlight the limitations of the study and explain potential areas for improvement.

**Reply:**

We appreciate this important comment. As you rightly pointed out, the prediction performance of the proposed method can deteriorate in the presence of strongly non-stationary epidemic patterns. In particular, COVID-19 does not exhibit the same level of periodicity as influenza. The distribution of cases can change abruptly due to factors such as viral mutations and interventions by individuals or governments, which makes handling non-stationarity even more critical.

In this study, we adopted a simple setting in which the dataset was partitioned based on known change points associated with viral variants (Delta to Omicron) and only past confirmed case numbers were used as input data. While this allowed us to evaluate the method under relatively controlled conditions, we acknowledge that addressing non-stationarity more explicitly is a key challenge for future work. We believe that developing models that are more robust to non-stationary patterns and exploring the use of external data sources that can help detect such changes will be important directions moving forward.

We have added a discussion of these limitations and potential directions for improvement in the revised manuscript.

**Changes in the manuscript:**

Information added in the manuscript: Discussion section, rows 410-420.

2. Lines 86–87: The key assumption for predicting time-series data is that there is no regime change in the dataset. Once the data becomes non-stationary, forward predictions become challenging. The authors should discuss how to detect regime or pattern changes.

**Reply:**

We appreciate your valuable input. As you pointed out, stationarity is a key concept in time-series forecasting. Infectious diseases such as COVID-19 are strongly influenced by changes in viral strains and societal responses, often resulting in non-stationary patterns. In general, even neural network models can be sensitive to sudden trend shifts, and traditional linear models like ARMA, which assume strong stationarity, face even greater limitations under such conditions. Therefore, it is desirable to detect change points and fit models within intervals that exhibit stationarity. In response to your comment, we have added references to literature that address the detection of unknown change points and a discussion on non-stationarity and change point detection. Although our study does not explicitly handle non-stationarity, as noted in the limitations section, we believe this addition helps enhance the understanding of its implications and potential approaches for dealing with such change points in time-series data.

**Changes in the manuscript:**

Information added in the manuscript: Related works section, rows 94-99.

3. Line 108: The integration of deep learning models (e.g., GNN) with epidemiological domain models has been explored by previous studies. The authors should clearly highlight the key methodological contributions of this work.

**Reply:**

Thank you very much for your insightful comment. We believe our study offers two methodological contributions that differentiate it from existing models integrating epidemiological domain knowledge.

First, while many existing models require not only the number of new infections but also the number of infectious individuals at each time point as input data, our model does not rely on such auxiliary data. Getting the number of infectious individuals typically requires information on recovered cases, which are often unavailable or underreported, limiting the applicability of existing models. In contrast, our approach estimates the number of infectious individuals internally using an estimation formula naturally derived from a reformulation of the SIR equations. This allows our model to operate solely on new infection data, making it more practical and widely applicable.

Second, while prior studies often focus on graph-based representations and employ graph representation learning with GNNs to capture spatial dependencies, our model adopts a simpler spatio-temporal processing approach using fully connected layers and a spatial embedding matrix. Although graph representation learning with GNNs is powerful, their complex network architectures can lead to increased computational costs and potential degradation in predictive performance. Our study demonstrates that accurate epidemic forecasting can be achieved without relying on such complex graph-based structures.

Regarding the first point, our explanation was insufficient, so we have revised the manuscript to provide a clearer and more detailed description.

**Changes in the manuscript:**

Description revised in the manuscript: Related works section, rows 124-132.

4. Line 146: How is the dimension of the latent space determined? The authors should also discuss the sensitivity of the results to this dimension.

**Reply:**

Thank you very much for the constructive comment. In our study, we manually explored several candidate values for the dimension of the latent space. Following the comment, we conducted experiments to assess the sensitivity of the model’s performance to the parameter and added the results as Fig 3.

**Fig 3. Sensitivity analysis results of the hidden dimension *D* across each dataset (*T_in_* , *T_out_* = 28).**

Our results showed that while the overall conclusions of the paper remain robust, the prediction accuracy can be affected by the choice of dimension. Specifically, a dimension that is too small may lead to reduced accuracy due to limited expressiveness, whereas a dimension that is too large may cause overfitting and degrade performance. Based on these findings, selecting a moderate latent space dimension is recommended to balance expressiveness and generalization. We have added this discussion to the revised manuscript.

**Changes in the manuscript:**

Information added in the manuscript: Experimental study section, rows 348-354.

5. Line 227: The data is pre-processed to remove seasonality. In practice, it is very difficult to detect periodicity in the early stages of prediction. The authors should elaborate on how this was handled.

**Reply:**

Thank you for your valuable comment. As you rightly pointed out, identifying periodicity in actual infection trends during the early stages of prediction is indeed challenging. However, the seasonality we removed was intended to address the effects of reporting practices by local governments and medical institutions, rather than the inherent periodicity of the infection itself.

Specifically, many medical institutions are typically closed on weekends, which often leads to a decrease in reported cases during those days. Additionally, delays in reporting due to system-related issues may result in data being aggregated and recorded on specific weekdays. These weekday-related fluctuations, stemming from the reporting system rather than the actual infection dynamics, can be anticipated even in the early stages of prediction.

To eliminate such artificial effects unrelated to the true infection trends, we applied a 7-day moving average—a commonly used method in epidemiological analyses. We have now added a clarification in the manuscript to better explain this point.

**Changes in the manuscript:**

Description revised in the manuscript: Experimental study section, rows 254-257.

6. The following references should be cited to strengthen the study’s context and relevance:

- Time series analysis and gated recurrent neural network model for predicting landslide displacements
- Ensemble learning of soil–water characteristic curve for unsaturated seepage using physics-informed neural networks
- Interpretation of spatio-temporal variation of precipitation from spatially sparse measurements using Bayesian compressive sensing (BCS).

**Reply:**

Thank you very much for your valuable suggestions. Regarding the first reference, we have cited it as an example of taking periodicity into consideration. As for the second reference, we have cited it in the manuscript to highlight the application of physics-informed neural networks (PINNs) in other domains. The third reference has been cited as an example of a method that effectively handles non-stationarity.

**Changes in the manuscript:**

Information added in the manuscript: Introduction section, rows 52-53, 99-100, 424-425
